# A special issue of *Essays in Biochemistry* on AMPK and AMPK-related kinases

**DOI:** 10.1042/EBC20240038

**Published:** 2024-11-18

**Authors:** Ian P. Salt, David Carling

**Affiliations:** 1 School of Molecular Biosciences, College of Medical, Veterinary and Life Sciences, University of Glasgow, Glasgow G12 8QQ, U.K.; 2 MRC Laboratory of Medical Sciences, Hammersmith Hospital Campus, Imperial College London, London W12 0HS, U.K.; 3 Institute of Clinical Sciences, Imperial College London, London W12 0NN, U.K.

**Keywords:** AMPK, LKB1, metabolic regulation

## Abstract

In eukaryotic cells, AMP-activated protein kinase (AMPK) plays a central role in responding to nutrient limitation by switching-off ATP-consuming (anabolic) pathways and switching-on ATP generating (catabolic) pathways. Over the last 30 years or so, a considerable body of research has been carried out that has provided us with a wealth of knowledge regarding the regulation and role of AMPK. Despite this, there is still much to learn about AMPK and the field remains highly active, with many groups around the world continuing to explore new roles for AMPK, providing insight into its biological function. This review series was inspired by recent AMPK-focused meetings in Scotland (2022) and Australia (2023) and draws on some of the research presented at those meetings.

The aim of this review series is to provide a broad overview of some of the more recent developments within the AMP-activated protein kinase (AMPK) field. We have included topics that cover the biological role of AMPK in integrating diverse signals upstream of AMPK with downstream cellular responses, together with technical advances used for monitoring AMPK expression and activity in cells. Whilst research on AMPK has tended to dominate the field, the AMPK-related kinases (ARKs) have emerged as important signal transducers of cell function, and the series includes a review on two of these kinases, NUAK1 and NUAK2.

The complexity and heterogeneity of cellular metabolism between different tissues and within tissues themselves impacts their function. This metabolic plasticity and variety within cells and tissues needs to be accounted for and Abbate et al. [1] discuss and compare methods and approaches for the detection of these variations including single cell RNA sequencing, mass spectrometry imaging as well as biosensors for metabolites and AMPK.

In a complementary article, Rider et al. [2] examine the use of mass spectrometry to quantify AMPK subunit isoform levels, identify AMPK substrates and interrogate mechanisms of AMPK activation. They discuss the challenges ahead concerning interpretation of big data sets and the potential for mass spectrometry-based single cell proteomics for targeted phosphoproteomics.

AMPK is activated by phosphorylation of AMPKα at Thr172 on the α subunit of the kinase when energy charge is reduced, and LKB1 (liver kinase B1) was the first upstream kinase to be identified in this cascade [3,4]. The same year, LKB1 orthologues were shown to similarly phosphorylate and activate the yeast AMPKα orthologue Snf1p at the equivalent site Thr210 [5]. In addition to AMPK, LKB1 phosphorylates and activates 12 other kinases termed ARKs [6]. The functions of these ARKs are less well-characterised compared with AMPK, yet our knowledge of their regulation and function is expanding rapidly. In this special issue, Skalka et al. review the structure and function of NUAK1 and NUAK2, one of the most well-characterised of the ARKs to date including emerging roles in redox and energy homeostasis [7].

Two years after the demonstration that LKB1 was an AMPKα Thr172 kinase, CAMKK2 (Ca^2+^-calmodulin-dependent protein kinase kinase-2) was identified as an alternative upstream activating kinase. CAMKK2 is stimulated by increases in Ca^2+^ levels, providing the first mechanism by which AMPK could be activated independent of energy charge [8–11]. Indeed, this activation by CAMKK2 had been reported previously using partially purified AMPK kinase fractions but was not identified as a physiological AMPK kinase at the time [12]. Since then, it has become apparent that CAMKK2 serves as an important link between calcium signalling and energy metabolism. McAloon et al. [13] provide an overview of the current knowledge concerning the regulation and roles of CAMKK2 including AMPK-dependent actions and apparently paradoxical roles of AMPK and CAMKK2 in liver function and prostate cancer.

TORC1 (target of rapamycin complex-1) integrates multiple signals to promote anabolism in response to growth factors, amino acids, and other signals. Among the earliest substrates defined for AMPK were TSC2 (tuberous sclerosis-2) and Raptor, which regulate and are a component of TORC1, respectively [14,15]. Identification of this suppression of TORC1 by AMPK was an exciting development as it showed how two key nutrient sensing kinase systems allowed the adaptation of cells to energy and nutritional status. In more recent years, it has become apparent that there are multiple cross-talk mechanisms between AMPK and TORC1 and Smiles et al. provide an overview of the current understanding of that cross-talk including direct and indirect phosphorylation, lysosomal activation, and control of autophagy [16].

As AMPK is a key regulator of glucose and lipid metabolism, there has been substantial interest in the roles of AMPK in the principal metabolic tissues, since activation of AMPK may act to improve insulin sensitivity and improve cardiovascular health. The final three essays focus on specific roles of AMPK in the maintenance of cardiometabolic health.

Frankish and Murphy [17] examine the role of the CBM (carbohydrate binding module) domain in the AMPKβ subunit, where *in vitro* evidence suggests that AMPK heterotrimers can bind to oligosaccharides including glycogen [18,19]. Given AMPK’s role in regulating muscle glycogen levels, this begs the question as to whether AMPK is associated with glycogen in muscle and whether this binding is regulated by or regulates AMPK. In their essay, Frankish and Murphy [17] review the evidence for an AMPK-glycogen interaction *in vivo* and highlight their recent work that demonstrates there is no association *in vivo* in rodent skeletal muscle.

The role of AMPK in adipose tissue is perhaps the least well-characterised of the metabolic tissues, although activation of AMPK has long been associated with suppression of lipogenesis (recently reviewed in [20]) and indirect and direct AMPK activators have been shown to suppress adipogenesis in cultured cell systems [21,22]. The review by Pollard examines the role of AMPK in adipose tissue with a particular focus on adipogenesis during healthy and pathological adipose tissue expansion [23], arguing that AMPK activation may be beneficial for healthy adipocyte differentiation and metabolism.

The final essay included in this special issue examines the role of AMPK in heart failure, which shares many risk factors with type 2 diabetes as well as other cardiovascular disorders. In their essay, Vanni et al. review the current understanding of AMPK in the context of heart failure, highlighting the recent evidence whereby AMPK regulates protein O-GlcNAcylation, which subsequently regulates cardiac metabolism and function [24]. Furthermore, they discuss whether there is reciprocal regulation between AMPK and O-GlcNAcylation and how AMPK activators may benefit heart failure.

We hope that this issue provides a useful resource for both non-experts as well as AMPK aficionados, that will inspire further research into the role of AMPK and ARKs, providing more new and exciting discoveries.

## Open Access

Open access for this article was enabled by the participation of Imperial College London in an all-inclusive *Read & Publish* agreement with Portland Press and the Biochemical Society under a transformative agreement with JISC.

## Abbreviations

AMPK: AMP-activated protein kinase
ARK: AMPK-related kinase
CAMKK2: Ca^2+^-calmodulin-dependent protein kinase kinase-2
CBM: carbohydrate binding module
LKB1: liver kinase B1
TORC1: target of rapamycin complex-1
TSC2: tuberous sclerosis 2
